# Individuals with obesity and COVID‐19: A global perspective on the epidemiology and biological relationships

**DOI:** 10.1111/obr.13128

**Published:** 2020-08-26

**Authors:** Barry M. Popkin, Shufa Du, William D. Green, Melinda A. Beck, Taghred Algaith, Christopher H. Herbst, Reem F. Alsukait, Mohammed Alluhidan, Nahar Alazemi, Meera Shekar

**Affiliations:** ^1^ Health, Nutrition and Population Global Practice The World Bank Washington, D.C. USA; ^2^ Department of Nutrition, Gillings School of Global Public Health University of North Carolina at Chapel Hill Chapel Hill North Carolina USA; ^3^ Carolina Population Center University of North Carolina at Chapel Hill Chapel Hill North Carolina USA; ^4^ Saudi Health Council Riyadh Kingdom of Saudi Arabia; ^5^ Community Health Sciences King Saud University Riyadh Kingdom of Saudi Arabia

**Keywords:** COVID‐19, individuals with obesity, meta‐analysis, vaccination

## Abstract

The linkage of individuals with obesity and COVID‐19 is controversial and lacks systematic reviews. After a systematic search of the Chinese and English language literature on COVID‐19, 75 studies were used to conduct a series of meta‐analyses on the relationship of individuals with obesity–COVID‐19 over the full spectrum from risk to mortality. A systematic review of the mechanistic pathways for COVID‐19 and individuals with obesity is presented. Pooled analysis show individuals with obesity were more at risk for COVID‐19 positive, >46.0% higher (OR = 1.46; 95% CI, 1.30–1.65; *p* < 0.0001); for hospitalization, 113% higher (OR = 2.13; 95% CI, 1.74–2.60; *p* < 0.0001); for ICU admission, 74% higher (OR = 1.74; 95% CI, 1.46–2.08); and for mortality, 48% increase in deaths (OR = 1.48; 95% CI, 1.22–1.80; *p* < 0.001). Mechanistic pathways for individuals with obesity are presented in depth for factors linked with COVID‐19 risk, severity and their potential for diminished therapeutic and prophylactic treatments among these individuals. Individuals with obesity are linked with large significant increases in morbidity and mortality from COVID‐19. There are many mechanisms that jointly explain this impact. A major concern is that vaccines will be less effective for the individuals with obesity.

## 1 INTRODUCTION

For persons with coronavirus disease 2019 (COVID‐19) caused by the severe acute respiratory syndrome coronavirus 2 (SARS‐CoV‐2), there appears to be a strong relationship between being an individual with overweight or obesity and the risks of hospitalization and needing treatment in intensive care units (ICUs). Emerging literature suggests that adults with obesity under the age of 60 are more likely to be hospitalized.^1^ The COVID‐19 pandemic has occurred at a time when the prevalence of individuals with overweight/obesity is increasing in virtually all countries globally. In fact, almost all countries today have a prevalence of individuals with overweight/obesity greater than 20%.^2^, ^3^, ^4^ To date, no country has experienced a reduction in the prevalence of individuals with overweight/obesity.

In addition, policy responses for mitigating COVID‐19 are creating major economic hardships. The COVID‐19 pandemic has brought to all countries the need to restrict movement, implement social distancing and impede economic activities across a broad spectrum of nonessential occupations. These adjustments have caused food system problems, including changes in food consumption and physical activity patterns, and remote telework environments that may exacerbate current trends in the prevalence of individuals with obesity, while another effect will be to increase the proportion food insecure and also those stunted and malnourished. These changes have long‐lasting implications beyond the mitigation of the current SARS‐CoV‐2 spread and may be detrimental to people's health.

The association between individuals with excessive body fat, especially visceral adipose tissue; individuals with obesity; major cardiometabolic problems, ranging from hypertension to cardiovascular disease to type 2 diabetes (T2D); and a number of cancers is strong.^5^, ^6^, ^7^, ^8^ The underlying metabolic and inflammatory factors of individuals with obesity also play a considerable role in the manifestation of severe lung diseases. Susceptibility to acute respiratory distress syndrome (ARDS), the primary cause of COVID‐19 mortality, is significantly greater among individuals with obesity.^9^ Importantly, being an individual with obesity independently increases the risk of influenza morbidity and mortality,^10^ most likely through impairments in innate and adaptive immune responses.^11^ Potentially the vaccines developed to address COVID‐19 will be less effective for individuals with obesity due to a weakened immune response.

In this paper, we first highlight the epidemiological data that provide insight into the relationship between being an individual with overweight/individuals with obesity and COVID‐19, undertaking when possible meta‐analyses of the published data. We provide an overview of the current understanding of how individuals with obesity affect the immunological and physiological response to SARS‐CoV‐2. We follow this with a discussion of the issues of income distribution, food insecurity and the major dietary shifts we are seeing globally. For the latter, we rely on reviews and reports from some key sources of industry sales data, as no solid primary data sources are available. Our discussion includes dietary and activity issues linked with COVID‐19 that might exacerbate individuals with obesity and some of the potential policies that can address this issue.

## 2 BACKGROUND: THE GLOBAL PREVALENCE OF INDIVIDUALS WITH OVERWEIGHT AND OBESITY

**FIGURE 1:**
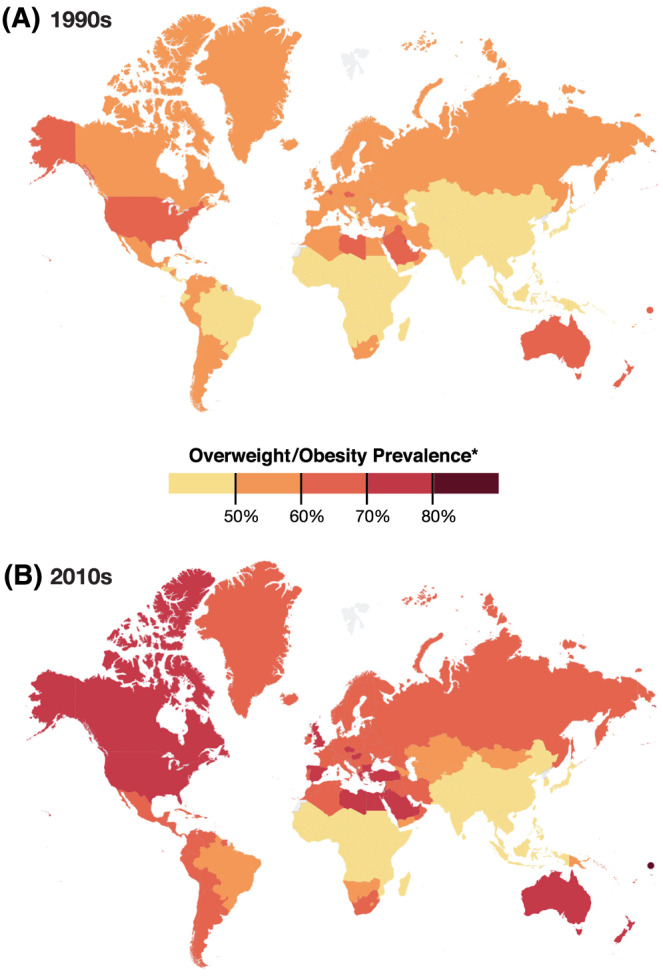
Prevalence of overweight and obesity based on 1990s and late 2010s weight and height data

The prevalence of individuals with overweight/obesity is at an all‐time high and is increasing across the globe. This is true not only in higher income countries but also in low‐ and middle‐income countries with high levels of undernutrition leading to the double burden of malnutrition.^4^, ^12^ Few low‐ and middle‐income countries have a prevalence of individuals with overweight/obesity less than 20% among their adult populations. Figure 1 shows a map of the world in the 1990s and the late 2010s.

A large proportion of the populations in higher income countries are overweight or obese. As Figure 1 shows, few higher income countries have adult populations with a prevalence of overweight/obese less than 70%. This prevalence is not declining in any country. In higher income countries, the prevalence of individuals with overweight/obesity was already high in the 1990s, and it has continued to increase. In fact, larger portions of their populations have become individuals with morbid obesity with body mass indexes (BMIs) over 35–40 kg m^−2^. In low‐ and middle‐income countries outside of Latin America and a number of small islands, the growth in individuals with overweight/obesity has occurred primarily in the past several decades from 1990 to 2020 which we and many others have documented.^2^, ^3^, ^13^, ^14^, ^15^, ^16^ Further, evidence shows that >70% of the individuals with overweight/obesity live in low‐ or middle‐income countries, and as country economies grow, the burden of individuals with obesity shifts to the poor.^17^, ^18^, ^19^ In the Middle East and Latin America, the prevalence of rates of individuals with obesity are among the highest in the world.

Two related factors are equally important. First, we are finding that much of the BMI increase accompanies an increase in central adiposity proxied by waist circumference at all ages compared with the amount of such adiposity one or two decades earlier.^20^, ^21^, ^22^ Second, across the globe, the economically poor are more prone to develop obesity than are the rich.^17^, ^18^, ^19^, ^23^

## 3 EPIDEMIOLOGICAL RELATIONSHIPS: INDIVIDUALS WITH OVERWEIGHT AND OBESITY AND COVID‐19

This review study is exempted from IRB review, and there was no public or patient involvement.

### 3.1 Literature retrieval

We examined PubMed, Google Scholar, MedRxiv, BioRxiv, Wanfang (for Chinese literature) and other literature search engines (e.g., China National Knowledge Infrastructure Data and ICNARC) to systematically review all publications in Chinese or English that include data on COVID‐19 and BMI or individuals with obesity. We briefly reviewed the abstracts and results and located 75 publications available by 15 July 2020 that presented data on the BMIs or BMI categories of diagnosed COVID‐19 patients. We excluded literature in other languages, as we read Chinese and English only. All of our authors performed the literature searches and reviews. Table S1 presents the search terms.

### 3.2 Study characteristics

**FIGURE 2:**
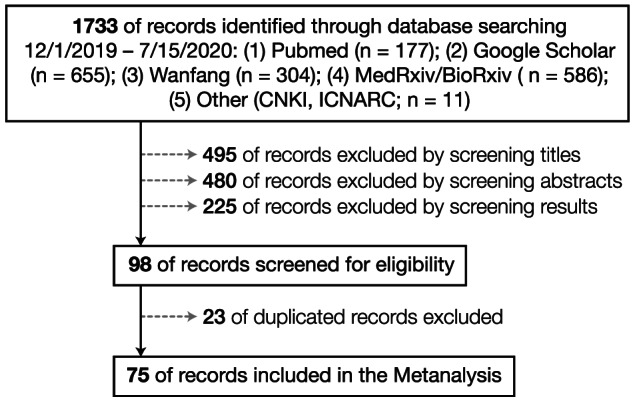
Flowchart for meta‐analysis of the obesity–COVID‐19 relationship

We found 1733 studies, 75 of which provided data we could use in this review (Figure 2). All were conducted between January and June 2020, including five case–control studies, 33 retrospective or prospective cohort studies and 37 observational cross‐sectional studies. Sample sizes varied from 24 to 109 367 diagnosed patients in more than 10 countries in Asia, Europe and North and South America. In total, we included 399 461 diagnosed patients in this study, about 55% of whom were male. Table S2 presents detailed demographic data from the studies we used, including a few studies that had inadequate data for use in the meta‐analysis. We used STATA (version 16, College Station, TX) to perform all random‐effects meta‐analysis and used residual maximum likelihood to fit all models.^24^

### 3.3 Being an individual with obesity and the risk of COVID‐19

**FIGURE 3:**
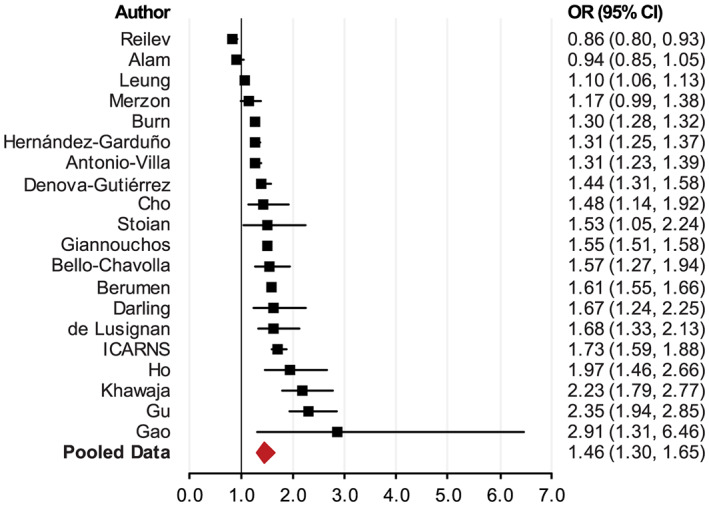
Meta‐analysis of the association between individuals with obesity and the risk of testing positive for COVID‐19

We identified 20 studies that assessed the association between individuals with obesity and COVID‐19, all but two of which showed that individuals with obesity significantly increase the risk of COVID‐19 (Table S3).^25^, ^26^, ^27^, ^28^, ^29^, ^30^, ^31^, ^32^, ^33^, ^34^, ^35^, ^36^, ^37^, ^38^, ^39^, ^40^, ^41^, ^42^, ^43^ One study in Denmark showed that the prevalence of overweight and individuals with obesity was lower in SARS‐CoV‐2 positive cases than SARS‐CoV‐2 test‐negative individuals (8.6% vs. 9.9%).^44^ The results may be biased because body weight status was determined at hospital discharge. A study used U.K. Biobank data (*n* = 285 817) to show that overweight increased the risk of COVID‐19 by 44.0% (relative risk [RR] = 1.44; 95% CI, 1.08–1.92; *p* = 0.0100) and individuals with obesity almost doubled the risk (RR = 1.97; 95% CI, 1.46–2.65; *p* < 0.0001), adjusted for age, sex, ethnicity and socio‐economic deprivation as measured by unemployment, assets and household density.^32^ The authors tested only a small portion of individuals (0.5%) for COVID‐19, a key limitation of this study. A better way to calculate OR for this study is to compare the odds between subjects who tested positive and those who tested negative. Our pooled data analysis showed that the odds of individuals with obesity being COVID‐19 positive were 46.0% (OR = 1.46; 95% CI, 1.30–1.65; *p* < 0.0001) higher than those of individuals who were not obese (Figure 3).

### 3.4 Being an individual with obesity and COVID‐19 illness severity

**FIGURE 4:**
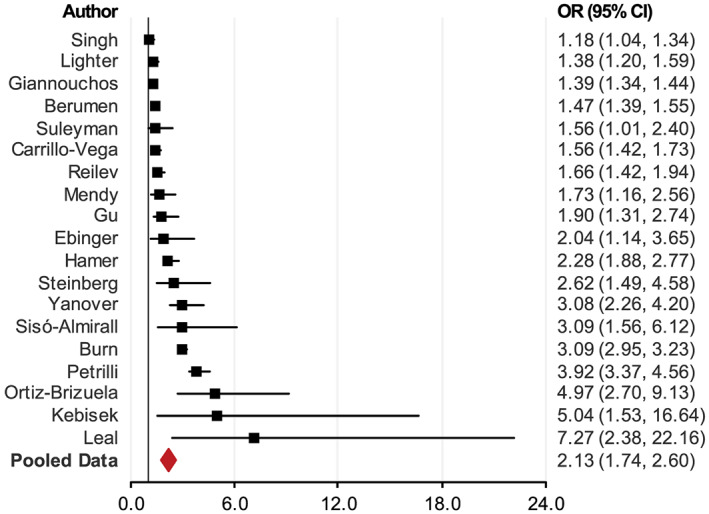
Meta‐analysis of the association between individuals with obesity and the risk of hospitalization with COVID‐19

Being an individual with obesity increases the odds of COVID‐19 patients being hospitalized. Among diagnosed COVID‐19 patients, the prevalence of individuals with obesity in hospitalized patients was much higher than that in nonhospitalized patients. For example, a report that included 5700 patients with obesity in New York City^45^ showed that 41.7% of COVID‐19 hospitalized patients were individuals with obesity, whereas the average prevalence of individuals with obesity in New York City was 22.0%.^46^ Many studies reported COVID‐19 hospitalizations, but only a few reported the relationship between individuals with obesity and hospitalization. We identified 19 studies that examined the relationship and included them in this analysis.^1^, ^28^, ^38^, ^40^, ^44^, ^47^, ^48^, ^49^, ^50^, ^51^, ^52^, ^53^, ^54^, ^55^, ^56^, ^57^, ^58^ Table S4 presents the results^1^, ^45^, ^47^, ^48^, ^59^, ^60^; all showed a significantly higher prevalence of individuals with obesity among hospitalized patients than among patients not hospitalized or the general population. The pooled OR was 2.13 (95% CI, 1.74–2.60; *p* < 0.0001) (Figure 4).

Among patients with symptoms, those with severe or critical conditions had much higher BMIs and individuals with obesity prevalence than the normal population or patients who were COVID‐19 negative.^32^, ^61^, ^62^, ^63^, ^64^, ^65^, ^66^, ^67^, ^68^, ^69^, ^70^ Two studies showed that the odds of having COVID‐19 increased by 30% (OR = 1.30; 95% CI, 1.09–1.54; *p* = 0.0030)^61^ and by 38% (OR = 1.38; *p* < 0.0001),^32^ respectively, among the individuals with obesity (Table 1).

**TABLE 1:** Body mass index (BMI) (kg m^−2^) distributions among COVID‐19 patients (mean with 95% CI or median with interquartile range)

First author	*N*	Mild	Critical	Average	National^71^, ^a^
Chen	145	23.2 (21.7–25.7)	24.8 (23.1,27.0)	23.7 (21.7–27.0)	23.9
Peng	112	22.0 (20.0–24.0)	25.5 (23.0–27.5)	22.0 (20.0–25.0)	23.9
Liao	81	24.5 (22.3–27.7)	23.9 (20.0–27.3)	24.0 (21.5–27.3)	23.9
Wu	280	23.6 ± 3.2	25.8 ± 1.8	24.1 ± 3.0	23.9
Liu	30	22.0 ± 1.3	27.0 ± 2.5	22.7 ± 2.3	23.9
Li	182			24.8 ± 4.1	23.9
Bhatraju	24			33.2 ± 7.2	28.8
Simonnet	124			29.6 (26.4–36.4)	25.3
Argenziano	1000	28.6 (25.2–33.1)	29.4 (25.7–34.2)	28.6 (25.2–33.1)	25.3
Prats‐Uribe	1039			29.2 ± 5.5	27.4
Raisi‐Estabragh	669			28.2 ± 6.3	26.7
Ho	340			29.0 ± 5.3	27.3

^a^ The National mean BMI data come from the country of the study.

**FIGURE 5:**
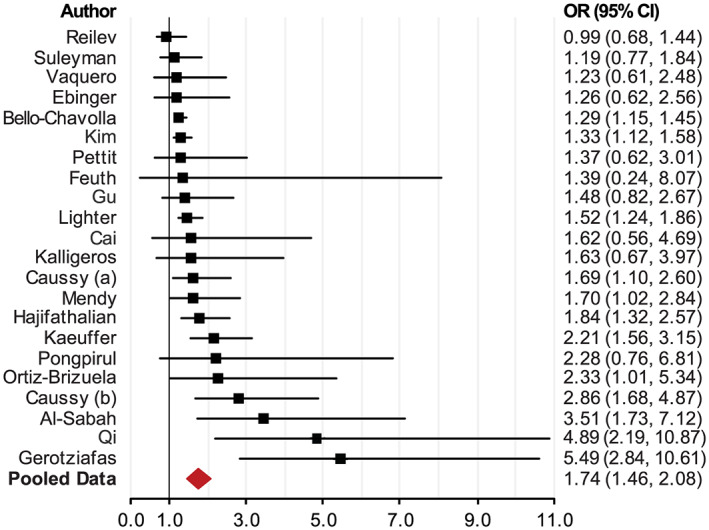
Meta‐analysis of the association between individuals with obesity and the risk of being placed in an intensive care unit (ICU)

All studies reported that among those diagnosed, patients with obesity were more likely to be admitted to ICUs. * However, the effect sizes in the studies with smaller sample sizes were not statistically significant.^48^, ^72^, ^73^ In the studies that found that being an individual with obesity did not significantly increase the odds of being admitted to the ICU, individuals with morbid obesity (defined as BMI ≥ 35) did significantly increase the odds of ICU admittance. Our pooled data (from 22 studies) showed that individuals with obesity increased the odds of being admitted to the ICU by 74% (OR = 1.68; 95% CI, 1.46–2.08; *p* < 0.0001) (Figure 5 and Table S5).

**FIGURE 6:**
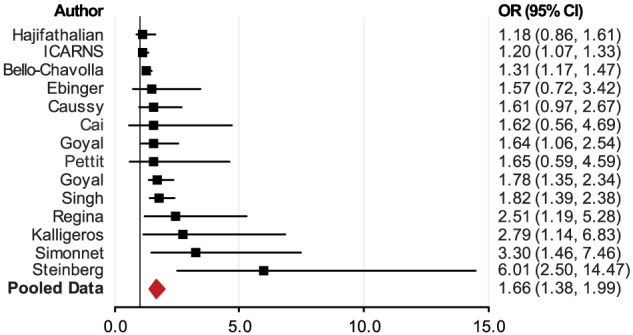
Meta‐analysis of the association between individuals with obesity and the risk of administration of invasive mechanical ventilation (IMV)

Reports that had smaller sample sizes from the United Kingdom and some other countries showed that patients with obesity had higher but insignificant odds of invasive mechanical ventilation (IMV) than patients without obesity.^48^, ^72^, ^86^ Reports from Mexico and some U.S. cities showed significantly higher odds of IMV in patients with obesity than in patients without obesity. † The pooled data (from 14 studies) showed a 66% increase in IMV in patients with obesity (OR = 1.66; 95% CI, 1.38–1.99; *p* < 0.0001) (Figure 6 and Table S6).

### 3.5 Being an individual with obesity and COVID‐19 prognosis

**FIGURE 7:**
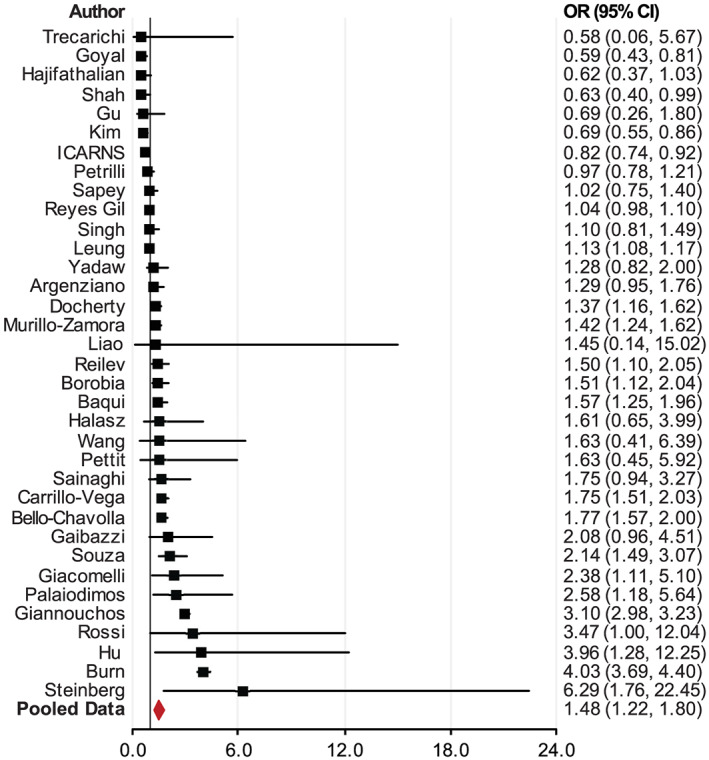
Meta‐analysis of the association between individuals with obesity and mortality for individuals with COVID‐19

The association between obesity and COVID‐19 prognosis is complex, because patients discharged from ICUs may be still hospitalized or deceased later. For example, 4.5% died after they were discharged from ICU; 11.5% remained in the hospital after leaving the ICU in one study.^31^ A few studies showed that individuals with obesity may decrease in‐hospital mortality.^31^, ^75^, ^87^, ^89^ Some studies showed that obesity may insignificantly decrease^41^, ^47^, ^81^, ^90^ or increase^55^, ^67^, ^68^, ^84^, ^91^, ^92^, ^93^, ^94^, ^95^, ^96^ the odds of death among individuals with obesity. The majority of studies showed that obesity significantly increased the odds of death among COVID‐19 patients with obesity. The pooled data (from 35 studies) showed that patients with obesity were more likely to have unfavourable outcomes with a 48% increase in deaths (OR = 1.48; 95% CI, 1.22–1.80; *p* < 0.001) (Figure 7 and Table S7). ‡ We excluded two studies that had very large OR and very wide 95% CI, one study in China (OR = 32.08; 95% CI, 6.73–153)^65^ and one in Nevada (OR = 10.55; 95% CI, 1.07–104.45),^106^ from Figure 7, but included them in the meta‐analysis.

## 4 WHY ARE INDIVIDUALS WITH OBESITY AT SERIOUS RISK FOR COVID‐19?

**FIGURE 8:**
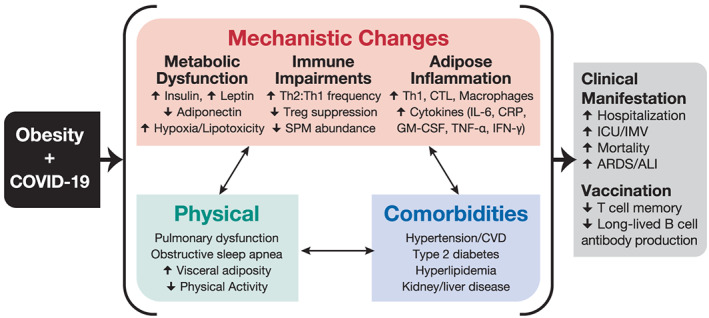
Clinical manifestations and mechanisms for COVID‐19 risk in individuals with obesity

Being an individual with obesity is associated with numerous underlying risk factors for COVID‐19, including hypertension, dyslipidaemia, type 2 diabetes (T2D) and chronic kidney or liver disease. Coronaviruses are typically not associated with severe disease and were mostly thought to cause only mild respiratory infections until the emergence of the 2002 severe acute respiratory syndrome coronavirus (SARS‐CoV) in Guangdong, China. The SARS‐CoV outbreak was ultimately contained thanks to its low viral load within the beginning stages of symptom onset, allowing time for identification and isolation of infected individuals.^107^ The 2009 influenza pandemic, caused by an outbreak of the upper respiratory influenza A H1N1 virus, identified individuals with obesity as an independent risk factor for severe influenza morbidity and mortality.^10^ Subsequently, emergence of the Middle East respiratory syndrome coronavirus (MERS‐CoV) in 2012 exhibited high prevalence among individuals with obesity.^108^ The growing evidence detailed above demonstrates that obesity increases the risks of hospitalization, severity and in some cases death with viral respiratory infections, increasing the likelihood that obesity may also independently increase the risk for COVID‐19, another respiratory viral disease. Several reports summarize the current understanding of the pathogenicity and immune response to SARS‐CoV‐2 based on available data from animal and human studies.^109^, ^110^ Importantly, the mechanism(s) responsible for greater COVID‐19 severity in individuals with obesity remains unknown. However, insights from other viral infections, like influenza, and epidemiological evidence offer some understanding of how being an individual with obesity increases the risk of COVID‐19 severity (Figure 8). Considering the exponential rise in the prevalence of individuals with obesity, understanding how being an individual with obesity increases the risk for severe COVID‐19 is critical to ensure appropriate interventional and prophylactic therapies against this novel coronavirus.

Figure 8 shows the effects of the physiological consequences of obesity on COVID‐19 clinical outcomes. These factors may also influence a vaccine response in individuals with obesity. There are other underlying factors—individual, household and community that impact significantly how we eat, drink and move, and we do not address their indirect impacts on COVID‐19.

### 4.1 Individuals with obesity's metabolic and physiological impairments linked to COVID‐19

Being an individual with obesity is a major risk factor for severe cases of certain infectious diseases, like influenza, hepatitis and nosocomial infections.^111^, ^112^ However, other infections, like tuberculosis, community‐acquired pneumonia and sepsis, have more favourable clinical outcomes in adults with obesity compared with lean adults.^113^ This supports the ‘obesity paradox’ hypothesis, where underlying characteristics of individuals with obesity influence the physiological response to infection. As with influenza infections, being an individual with obesity appears to increase COVID‐19 severity. Obesity is inherently a metabolic disease characterized by alterations in systemic metabolism, including insulin resistance, elevated serum glucose, altered adipokines (e.g., increased leptin and decreased adiponectin) and chronic low‐grade inflammation.^114^, ^115^ Strong evidence demonstrates how hormone and nutrient dysregulation in individuals with obesity can impair the response to infection.

Hyperglycaemia, a key hallmark of T2D, is highly associated with individuals with obesity. Importantly, uncontrolled serum glucose has been shown to significantly increase COVID‐19 mortality.^116^ During times of infection, uncontrolled serum glucose can impair immune cell function either directly or indirectly via generation of oxidants and glycation products.^117^ Similarly, both insulin and leptin signalling are critical in the inflammatory effector response of T cells by up‐regulating cellular glycolysis,^118^, ^119^ supporting the production of effector cytokines such as IFN‐γ and TNF‐α. These metabolic factors combine to influence immune cell metabolism,^120^ which dictates the functional response to pathogens, such as SARS‐CoV‐2.

Dietary consumption of fatty acids can also influence inflammatory responses. Prostaglandins, the derivatives of long chain fatty acids, are acute phase pyrogens that initiate the local inflammatory response during infection. Omega‐3 polyunsaturated fatty acids can induce anti‐inflammatory responses through cyclooxygenase (COX) activity, whereas omega‐6 fatty acids mediate the pro‐inflammatory COX production of prostaglandins.^121^, ^122^, ^123^ Current dietary intakes favour omega‐6 fatty acids over omega‐3s, with U.S. consumption currently in a 10:1 ratio due to the widespread consumption of vegetable oils.^124^ Fatty acid derivatives can directly influence COVID‐19 in individuals with obesity. Preclinical data suggest a role for fatty acid derived pro‐resolving lipid mediators, as they can be deficient in individuals with obesity and thus are not able to appropriately resolve inflammatory responses during infection.^125^

Other fatty acids, such as cholesterol, are essential in the spread of enveloped RNA viruses, like respiratory syncytial viruses and influenza. SARS‐CoV, the nearest relative to SARS‐CoV‐2, uses cholesterol to facilitate viral budding following S protein binding of cellular ACE2 receptors, allowing the spread to neighbouring cells. Depletion of cholesterol in ACE2 expressing cells results in markedly reduced viral S protein binding.^126^ Being an individual with obesity also increases the risk of COVID‐19 severity among patients with metabolic associated fatty liver disease, where adults with obesity had a greater than six fold higher risk for severe COVID‐19 regardless of age, sex or comorbidities, such as hypertension, diabetes and dyslipidaemia.^127^

Physical features of individuals with obesity also likely increase COVID‐19 severity and risk. Obstructive sleep apnoea and other respiratory dysfunctions in the individuals with obesity often increase risk of hypoventilation‐associated pneumonia, pulmonary hypertension and cardiac stress.^128^ Large waist circumference and greater body mass increase the difficulty of care in hospital settings for supportive therapies, such as intubation, mask ventilation and prone positioning to help reduce abdominal tension and increase diaphragm capacity.^129^ Thus, the prognoses of COVID‐19 patients with obesity may be complicated by the increased clinical care burden among this already vulnerable group.

### 4.2 Being an individual with obesity impairs the immune response to SARS‐CoV‐2

Being an individual with obesity has modulatory effects on key immune cell populations critical in the response to SARS‐CoV‐2. Specifically, increased BMI is associated with greater frequency of the anti‐inflammatory CD4 T cell subsets Th2 and T regulatory cells.^130^ Increased anti‐inflammatory cells may inhibit the ability to reduce the infection, as inflammatory responses are needed to control viral spread. Regulatory T cells (Tregs) primarily resolve immune cell mediated inflammation following infection. Tregs from hyperinsulinaemic mice with obesity have reduced interleukin 10 (IL‐10) production^131^ and, despite being in higher abundance in the lungs during influenza infections, are 40% less suppressive.^132^ Functional responses to RNA viruses, like SARS‐CoV‐2, rely on type 1 inflammatory responses by Th1 cells for protection with optimal anti‐inflammatory Treg responses for immune resolution following infection. Severe cases of individuals with influenza and COVID‐19 share remarkably similar reliance on type I interferon activation, with TNF/IL‐1β‐driven inflammation present in severe but not mild cases.^133^ Any imbalance in these T cell subsets or functions is likely to impair the immune response to SARS‐CoV‐2.

A further imbalance in immune cell subsets occurs with accumulation of pro‐inflammatory cells, including macrophages, dendritic cells, cytotoxic T cells and Th1 cells, in the adipose tissue of obese individuals. This influx of immune cells contributes to the development of insulin resistance and chronic inflammation.^134^ These pro‐inflammatory immune cells along with hypertrophic adipocytes are responsible for increased serum inflammatory cytokines, such as IL‐6, C‐reactive protein and type I and type III interferons.^135^, ^136^ This immune phenotype can be further distinguished between nondiabetic and people with diabetes and obesity through increased Th17 inflammation driven by impaired immune cell oxidation of fatty acid metabolites.^137^, ^138^

These changes in systemic immune cell populations and their accumulation in adipose tissue have been proposed as key mediators of COVID‐19 severity in individuals with obesity.^139^ Recently, mice with obesity infected with lymphocytic choriomeningitis virus (LCMV) were shown to have increased LCMV viral titres and LCMV‐specific immune cells in white adipose tissue, which upon secondary infection resulted in greater inflammation and mortality in mice with obesity compared with mice that are lean.^140^ Accumulation of adipocytes and adipocyte‐like cells can increase immune activation and cytokine production during coronavirus infection.^141^ In addition to being nutrient‐rich storage pools, lipid accumulation and adipocyte hypertrophy might be an immune reservoir that in individuals with obesity becomes saturated with pro‐inflammatory immune cell subsets.

Alterations in immune cell frequencies in individuals with obesity have been proposed for SARS‐CoV‐2 severity, which uses the angiotensin‐converting enzyme 2 (ACE2) for viral entry and is highly expressed in vascular tissues like the lungs and adipose tissue.^141^ Viral entry via ACE2 cleavage by the serine protease TMRPSS2 spike protein allows viral replication not only in the respiratory tract but also in other tissues expressing ACE2, including the intestinal enterocytes, liver, heart and kidneys.^109^, ^142^ This mechanism is thought to drive increased incidence of ischemic and coagulopathy conditions in COVID‐19 patients.

### 4.3 Inflammatory considerations of COVID‐19 in individuals with obesity

ARDS and acute lung injury (ALI) are two of the primary causes of morbidity and mortality among adults infected with SARS‐CoV‐2.^143^ Presentation of ARDS and ALI is characterized by respiratory failure due to excessive pro‐inflammatory cytokine production. This inflammatory state leads to extensive lung damage, hypoxemic respiratory failure regardless of oxygen administration and pulmonary oedema not caused by congestive heart failure.^144^ Patients who develop ARDS are typically administered mechanical ventilation with positive end‐expiratory pressure and high FiO_2_. Currently, adults with obesity infected with SARS‐CoV‐2 have higher burdens of mechanical respiratory therapy support and ARDS development.^66^

Gong et al. previously demonstrated that, compared with lean adults (BMI 18.5–24.9), adults with obesity are more likely to develop ARDS.^9^ A 2016 meta‐analysis investigating how BMI influences ARDS/ALI outcomes demonstrated significantly lower ARDS‐related mortality in adults with obesity compared with lean adults despite confirming greater odds for developing ARDS.^145^ However, a retrospective multicentre study in Wuhan, China, found higher ARDS‐related mortality among COVID‐19 patients, which was predicted by elevated serum IL‐6.^143^ Similarly, elevated IL‐6 is a hallmark of severe SARS‐CoV,^146^ MERS‐CoV^147^ and pandemic H1N1 influenza A viral infections.^148^ Additionally, severe COVID‐19 cases have been associated with lymphopenia^149^ and lower expression of IFN‐γ by CD4 T cells.^150^ IFN‐γ is an important antiviral protein, and reduced production of this cytokine in response to influenza has been documented previously in both models of mice with obesity and human populations with obesity.^132^, ^151^

Men also experience a higher burden of COVID‐19 than women.^59^ Being a man with obesity increases aromatase activity, which can convert testosterone to estradiol.^152^ Oestrogen receptor signalling can subsequently down‐regulate IL‐6 expression through inhibition of NF‐κB,^153^ which has been shown to confer protective effects against influenza A virus in women through stimulation of neutrophil and virus‐specific CD8 T cell responses.^154^ Interestingly, however, men with obesity have impaired oestrogen receptor signalling, which leads to increased androgenic hormones and elevated oestrogen production from adipose tissue.^155^ Recently, androgen depletion therapy has been shown to protect against COVID‐19 in male prostate cancer patients.^156^ However, more information is needed to understand the mechanism of action of androgens and androgen depletion therapy. Nonetheless, adequate control of pro‐ and anti‐inflammatory responses during SARS‐CoV‐2 infections is critical to limit nonspecific tissue damage and subsequent development of ARDS, which has a higher burden among COVID‐19 cases with obesity.

### 4.4 Implications for treatment and vaccination strategies for being an individual with obesity

Obesity may also impair therapeutic treatments during COVID‐19 infections. ACE inhibitors, which are commonly used to treat hypertension, may increase COVID‐19 severity in T2D patients, especially those with poorly controlled blood glucose.^157^ While discontinuing use of ACE inhibitors is not advisable at this time due to offsetting cardiovascular benefits,^158^ current clinical trials are investigating mitigation of the spread of SARS‐CoV‐2 through inhibition of ACE2 binding. How these treatments in patients with obesity contribute to COVID‐19 severity, however, will be a key question in their overall effectiveness. The IL‐6 receptor (IL‐6R) antagonist tocilizumab may reduce IL‐6 signalling in severe COVID‐19 cases where cytokine release syndrome is a major factor of mortality.^159^ As noted above, chronic inflammation is a hallmark of individuals with obesity, which includes elevated levels of IL‐6. Preliminary data suggest tocilizumab treatment can reduce fever and oxygen requirement.^160^ However, subjects with obesity with chronically elevated IL‐6 may not benefit from acute treatment. Dexamethasone, a corticosteroid commonly used for inflammatory treatment of arthritis, allergic reactions or other immune inflammatory disorders, has been shown in preliminary data to reduce mortality in severe COVID‐19 patients by 8–26%.^161^ These data from the RECOVERY trial provide evidence of reduced 28‐day mortality with treatment of 6‐mg dexamethasone over a consecutive 10‐day period during the symptomatic phase, resulting in inclusion of dexamethasone as a treatment option under the National Health Service COVID‐19 treatment protocol,^162^ joining remdesivir as the only approved treatment options for COVID‐19. There remains limited information on other treatments, such as statins, nonsteroidal anti‐inflammatory drugs and angiotensin receptor blockers, regarding their effectiveness against COVID‐19 in the individuals with obesity. Considering that almost all countries today have a prevalence of individuals with overweight/obesity greater than 20% and that in certain countries, such as the United States and the United Kingdom, two‐thirds of the population is overweight/obese, understanding how these therapies work in the host with obesity is critical information to determine their long‐term effectiveness.

Importantly, being an individual with obesity has also been shown to impair the development of immunological memory. Influenza vaccination in adults with and without obesity results in equivalent influenza‐specific antibody titres at 30 days post vaccination, but antibody titres wane significantly more in adults with obesity compared with adults who are lean at 1 year post vaccination.^163^ Compared with influenza‐vaccinated lean adults, vaccinated adults with obesity have impaired CD4 and CD8 T cell production of key inflammatory cytokines IFN‐γ and granzyme B.^151^ Adults with obesity also have two times greater odds of influenza or influenza‐like illness despite a robust antibody response.^164^ Preclinical evidence demonstrates that adjuvant vaccines confer less protection against influenza viruses in diet‐induced mice that are obese.^165^ Similar impairments in vaccine effectiveness have been reported individuals with obesity for tetanus,^166^ hepatitis A and B and rabies.^167^

Data from recovered COVID‐19 patients show greater than 95% of infected patients develop neutralizing antibodies against SARS‐CoV‐2. However, early evidence suggests a waning of antibody production over a period of weeks to months,^168^ suggesting vaccines strategies designed for antibody seroprotection may not have as long‐lasting effects. This fast decline in circulating neutralizing antibodies is more similar to common coronaviruses as opposed to SARS‐CoV, which has a longer sustained level of antibody titres of ~2 years.^169^ Promising data from multiple groups find cross‐reactive T cell responses in 70–100% of COVID‐19 patients.^170^, ^171^ Le Bert et al. showed 36 convalescent COVID‐19 patients all had CD4 and CD8 T cells capable of recognizing and responding to the NP protein of SARS‐CoV2. Importantly, they demonstrate presence of long‐lived memory T cells in 23 patients who recovered from SARS‐CoV.^172^ Several other key papers find T cell mediated immune responses to SARS‐CoV‐2 across cohorts, suggesting generation of memory T cell populations is critical for any future COVID‐19 vaccine.^173^ Unfortunately, as T cell responses have been shown to be impaired in individuals with obesity, this suggests that a future COVID‐19 vaccine may be less effective in an population with a high prevalence of individuals with obesity. Therefore, it is urgent that any vaccine trials and studies include BMI as a potential confounder for vaccine effectiveness and protection.

## 5 COVID‐19 ECONOMIC EFFECTS: HOW DOES THE PANDEMIC INDIRECTLY AFFECT OUR DIETS AND WEIGHT GAIN?

COVID‐19 has led not only to increased unemployment and income insecurity but to many changes in food supplies. Many aspects of food supply chains have been disrupted, and components of the food system focused on restaurants and hotels have lost their demand and are experiencing difficulty redirecting toward home consumption. Other key aspects of food chains, especially in low‐ and middle‐income countries have been completely disrupted with impacts varying by country and region. There is an expectation of a significant rise in stunting and adult thinness is expected, especially in South Asia, a few select other SE Asian countries (e.g., Indonesia) and much of sub‐Saharan Africa along with pockets of the poor in all other low‐ and middle‐income countries.^174^ The impact on not only malnutrition but increased food insecurity for the large proportion of lower income families is expected to be significant.^174^

One might suspect we would see a decline in obesity if the food insecurity impacts the individuals with overweight and obesity in many low‐ and middle‐income countries. This truly depends on how serious is the food insecurity and loss of income and how are diets shifted, if at all. We will see diet shifts in not only how we eat and drink but also how we move if inactivity grows greatly. If the diets shifts to increased consumption of refined carbohydrates, fried food and other unhealthy aspects of the traditional diet or to increased highly or ultraprocessed food we may experience increases in the prevalence of individuals with obesity. One can speculate but we truly do not know. Surveys on this topic are not published to date. Similarly studies in higher income countries suggest weight gains or no shift in weight.^175^ At the same time, some studies from higher income countries suggest potential increases in obesity.^175^, ^176^

While we do not have data on sales of ultraprocessed foods and beverages, many reports both from organizations monitoring food purchases and global company reports suggest that in higher and middle‐income countries access to fresh foods, especially fruits and vegetables is impacted due to breakdowns in local supply chains, and the demand for packaged processed food has increased, especially in the ready‐to‐eat and ‐drink categories.^8^, ^177^ These foods tend to be ultraprocessed and high in energy density, saturated fat, sodium and sugar. The attraction is partially that these foods require less storage and are highly palatable. In addition, they are relatively inexpensive due to the large economies of scale in their production. Particularly where costs loom greatly in food‐purchasing decisions, as among the lower income segments of the population, these cheaper products may be consumed in much greater quantities. However, ultraprocessed foods are a major contributor to obesity and other non‐communicable diseases (NCDs). The literature linking ultraprocessed foods with adverse health outcomes is large and consistent.^178^, ^179^, ^180^, ^181^, ^182^, ^183^, ^184^, ^185^, ^186^, ^187^, ^188^, ^189^, ^190^, ^191^, ^192^, ^193^, ^194^, ^195^

Additionally, the lockdown and fear of contact with the virus will likely have reduced walking and other movements among all age groups while enhancing sedentary living, TV and computer and video games. We would expect significant declines in energy expenditures from this combination of reduced movement and increased sedentary behaviours. Concurrently, the rapid increase in consumption of ultraprocessed foods and reduced energy expenditures in almost all low‐, middle‐ and high‐income countries are expected to heighten the risks of overweight, obesity and other NCDs.^196^

## 6 DISCUSSION AND POLICY IMPLICATIONS

It is clear that increasing prevalence of individuals with overweight/obesity among adults and the elderly is a major worldwide problem. Individual with overweight and obesity face a greater risk of severe consequences from COVID‐19, including hospitalization, intensive clinical care requirements and death. Moreover, individuals with obesity are likely to face reductions in the effectiveness of vaccines through mechanisms similar to those responsible for greater primary infection risk. Furthermore, it is quite possible that social distancing and stay‐at‐home policies may exacerbate adverse weight and health situations through their effects on dietary and physical activity patterns. Governments must consider actions to address not only long‐term economic issues but also diet quality during this and future pandemics to build resilience.

The immunological impairments from individuals with obesity demonstrate the convergence of chronic and infectious disease risks. They expose a large portion of the world population with overweight/obesity status to greater risk of pulmonary viral infections like COVID‐19. Given the expanding prevalence of individuals with overweight/obesity, it is imperative to consider the consequences of the related impaired immune responses during development of therapies and vaccines. Additional research is needed to understand the causal relationships. Limited information is available on how COVID‐19 is influenced by metabolic, hormonal or inflammatory factors, all of which have been previously shown to influence responses to infection in other disease contexts. The hidden factors of obesity, such as the potential divergence in the host microbiome, genetic or epigenetically inheritable traits or dietary patterns and insufficiencies in expanding populations with obesity, may elucidate the difference between severe and nonsevere COVID‐19 cases. Further, it is entirely possible that the current pandemic could unintentionally worsen NCDs in adults with overweight/obesity status.

COVID‐19 is an unparalleled event in modern human history. It has changed human lives and societies entirely. On the one hand, social distancing and stay‐at‐home policies have paused many economic activities and have created tremendous fiscal and health burdens for governments and individuals, especially the poor. These measures have increased consumption of unhealthy processed foods and have decreased physical activity. On the other hand, being an individual with overweight/obesity increase the risk of SARS‐CoV‐2 infection and worsen COVID‐19 outcomes, as discussed above. To date over 600 000 people have died from COVID‐19 globally with over 14 million total cases. The disease has directly or indirectly affected nearly every individual's life in countries all over the world. We need interdisciplinary collaborative efforts to tackle this disease. We also need to develop policies regarding infectious diseases to maintain a sustainable environment and healthy lifestyles.

As an aside, it is useful to note that China and several other Asian countries such as South Korea and Vietnam all saw limited impacts of COVID‐19 and all have very low prevalence of individuals with overweight and obesity. One might speculate that the reduced prevalence of individuals with obesity is linked with reduced risk and mortality for these countries, but there are way too many other factors to accept such speculation.

### 6.1 Policy implications

Vaccination remains the best protection against infectious diseases like COVID‐19. Therapeutics targeted at limiting viral replication or remediating complications of infection may help limit severe cases and moderately reduce mortality. Public health experts agree that viral spread will continue to cause tremendous health and economic problems until we reach vaccination and/or community‐acquired herd immunity. Current models project that intermittent times of social distancing and lockdown measures will be required until a viable vaccine can be widely produced,^197^ and these measures are likely to extend into the foreseeable future. This paper highlights another concern—that is, vaccines may not be as effective in individuals with overweight/obesity. Given the large prevalence of the world population that is composed of individuals with overweight/obesity, it is imperative that governments ensure that testing and research focus not only on the general efficacy of vaccines and therapeutics but also on how they will impact individuals with obesity.

Furthermore, we must carefully monitor and regulate the consumption of ultraprocessed foods and beverages through fiscal policies such as taxation and regulating marketing and promotion of such foods. If as expected this behaviour is increasing, it will exacerbate other health concerns, including risks of increased adiposity and major NCDs. When compounded with reduced physical activity and increased sedentary behaviour, the risk of increased adiposity is clearly an important concern. Finally, the poor around much of the globe also face increased hunger and with it the potential for elevated stunting and its consequences, including the long‐term risks of central visceral adiposity and many NCDs. Increasing hunger and stunting can have long‐term adverse impacts on health and well‐being in multiple ways, and major policies to mitigate this effect are critical when resources are available.

In addition to COVID‐19's critical economic constraints, its impacts on diets may pose lifelong risks to populations around the globe. Food habits developed during this period, particularly the intake of ultraprocessed foods, represent a major health risk. Exact policy prescriptions will be country specific, and clearly, the concerns for higher and middle‐income countries will differ from those of low‐income countries. NCD and individuals with obesity risks are far more predominant in the former, whereas the latter face high levels of the double burden of malnutrition, in which slow declines in stunting are likely to shift to increased stunting and wasting accompanied by rapid increases in individuals with obesity.

Creative policies to reduce consumption of ultraprocessed foods and increase consumption of healthier foods, such as legumes, selected whole grains, vegetables and fresh fruits, are important for all countries. A recent World Bank report suggests that the multipurpose Chilean model effectively administers multiple regulations and laws that reinforce each other and are impactful.^12^, ^198^, ^199^ It is quite that likely Chile's policies could significantly reduce the current growth in consumption of ultraprocessed foods. Moreover, a tax accompanying purchases of those foods would potentially increase fiscal space in countries suffering from the economic impacts of COVID‐19, albeit few countries have successfully allocated these resources for health or nutrition programmes. Some countries are finding ways to provide boxes of fresh vegetables and fruits to the elderly such as one programme in several Chilean cities^200^; however, most low‐ and middle‐income countries do not have the resources for such efforts though combined with taxation and marketing controls, such efforts would be more feasible. All countries need to consider how to enhance consumers' selections of healthy food options while reducing incentives to purchase ultraprocessed foods and beverages. To date, no country has combined these fiscal and regulatory policies. However, Israel's Chilean‐style warning labels and promotion of healthy eating comes closest.^201^

The COVID‐19 pandemic challenges all countries enormously. Our systems, institutions, health and welfare will feel the impacts for many years. The high prevalence of individuals with obesity exacerbates the threat to everyone's health, and the economic, social distancing and stay‐at‐home components compound the impacts. We will need creative solutions quickly to prevent undesirable dietary patterns and promote healthy eating, which is so critical to our future health and for building resilience against future threats.

## CONFLICT OF INTEREST

The authors declare that they have no competing interests.

## AUTHOR CONTRIBUTIONS

B.P., C.H., R.M., M.A., N.A. and T.A. conceptualized the study. S.D. led the meta‐analysis and B.P. helped. W.G. edited the mechanistic pathways work with M.B. B.P. drafted the introduction and discussion, and all co‐authors were involved in the rewrite and review for the final version.

## DATA AND MATERIALS AVAILABILITY

All data are presented in the paper, and all references are publicly available.

## Supporting information


**Table S1.** Search terms used in the literature retrieval
**Table S2.** Characteristics of participants and studies for all meta‐analyses
**Table S3.** Association between being obese and the risk of COVID‐19
**Table S4.** Prevalence of overweight/obesity and risk of hospitalized of COVID‐19 patients
**Table S5.** The association between obesity and ICU admission
**Table S6.** The association between obesity and IMV admission
**Table S7.** The association between obesity and prognosis of COVID‐19

## Footnotes

* References 1, 27, 31, 41, 44, 48, 54, 72, 73, 74, 75, 76, 77, 78, 79, 80, 81, 82, 83, 84, 85.

† References 27, 31, 55, 66, 73, 81, 87, 88.

‡ References 25, 27, 38, 40, 44, 47, 49, 57, 65, 97, 98, 99, 100, 101, 102, 103, 104, 105.
