# Supplementary material for: Individuals with obesity and COVID‐19: A global perspective on the epidemiology and biological relationships
Source: Obes Rev. 2020 Aug 26;21(11):e13128. doi: 10.1111/obr.13128 (PMC7461480; doi:10.1111/obr.13128)
Supplement: Supplementary file 1 — Table S1. Search terms used in the literature retrieval Table S2. Characteristics of participants and studies for all meta‐analyses Table S3. Association between being obese and the risk of COVID‐19 Table S4. Prevalence of overweight/obesity and risk of hospitalized of COVID‐19 patients Table S5. The association between obesity and ICU admission Table S6. The association between obesity and IMV admission Table S7. The association between obesity and prognosis of COVID‐19 [file OBR-21-0-s001.docx]

**Online Supplemental materials**

**Table 1. Search terms used in the literature retrieval**

| **Search engines** | **Search terms** |
| --- | --- |
| Pubmed, Google Scholar, MedRxiv, BioRvix, ICNARC | Covid + obesity, Covid + BMI, SARS-CoV-2 + obesity |
| Wanfang, CKNI | 新冠肺炎 + 肥胖, 新冠肺炎 + 体质指数, 肺炎, 肥胖 |

**Table 2. Characteristics of participants and studies for all metaanalyses**

| **Author** | **N** | **Country** | **Age (IQR or Mean, years)** | **% Female** | **Outcomes** | **BMI collected** | **Confounders adjusted** | **Type of study** |
| --- | --- | --- | --- | --- | --- | --- | --- | --- |
| Alam | 1,516 | Bangladesh | Adults, all ages | 46.1 | Incidence | Self-reported |  | Cross-sectional |
| Al-Sabah | 1,158 | Kuwait | 31.5-52.1 | 18.2 | ICU admission | Medical record | Age, gender | Cohort |
| Antonio-Villa | 10,925 | Mexico | 10.7 | 59.9 | Incidence | Self-reported |  | Cross-sectional |
| Antwi-Amoabeng | 172 | USA (NV) | 33.5-68 | 44.2 | Mortality | Medical record |  | Retrospective |
| Argenziano | 1,000 | USA (NY) | 50-75 | 40.4 | Hospitalization, ICU admission | Medical record |  | Retrospective |
| Baqui | 11,321 | Brazil | Adults, 18+ | 27.2 | Mortality | Medical record |  | Cross-sectional |
| Bello-Chavolla | 15,529 | Mexico | 46.6±15.5 | 42.2 | ICU and IMV admission, mortality | Medical record |  | Cross-sectional |
| Berumen | 31,522 | Mexico | 0+ | 41.5 | Hospitalization | Medical record |  | Case-control |
| Bhatraju | 24 | USA (WA) | 23-97 | 37.5 | ICU admission, mortality | Medical record |  | Cross-sectional |
| Borobia | 2,226 | Spain | 46-78 | 51.8 | Mortality | Medical record |  | Cohort |
| Burn | 109,367 | Spain | 36-61 | 59 | Incidence, hospitalization, mortality | Medical record |  | Cohort |
| Cai | 383 | China | 28-62 | 52 | Severity of disease | Medical record | Age, gender | Cross-sectional |
| Carrillo-Vega | 10,544 | Mexico | 48.2 ± 14.4 | 42.3 | Hospitalization, ICU admission, mortality | Medical record |  | Cross-sectional |
| Caussy | 291 | France | Not reported | Not reported | ICU admission | Medical record |  | Cohort |
| Caussy (a) | 110 | France (Lyon) | Not reported | Not reported | ICU admission | Medical record |  | Retrospective cohort |
| Caussy (b) | 124 | France (Lille) | Not reported | Not reported | ICU admission | Medical record |  | Retrospective cohort |
| Chen | 145 | China | 47.5±14.6 | 45.5 | Severity of disease | Medical record |  | Cross-sectional |
| Cho | 538 | UK | 40-86 | 44.1 | Incidence | Medical record |  | Cross-sectional |
| Darling | 580 | UK | 57.5±8.7 | 42.1 | Incidence | Medical record |  | Case-control |
| de Lusignan | 587 | UK | 0+ | 49.6 | Incidence | Medical record |  | Cross-sectional |
| Denova‐Gutiérrez | 3,844 | Mexico | 45.4 ± 15.8 | 42 | Incidence | Self-reported |  | Cross-sectional |
| Docherty | 16,749 | UK | 57-82 | 30.4 | Hospitalization | Medical record |  | Cohort |
| Ebinger | 442 | USA (CA) | 52.7 ± 19.7 | 42.1 | ICU admission | Medical record |  | Cross-sectional |
| Feuth | 28 | Finland | 47-72 | 46.4 | ICU admission | Medical record |  | Retrospective cohort |
| Gaibazzi | 279 | Italy | 60-80 | 39.4 | Mortality | Medical record |  | Cross-sectional |
| Gao | 150 | China | 48 | 37.3 | Severity of disease | Self-reported |  | Case-control |
| Gerotziafas | 310 | France | 19-95 | 39 | ICU admission | Medical record |  | Cohort |
| Giacomelli | 233 | Italy | 18-95 | 30.9 | Mortality | Medical record |  | Cohort |
| Giannouchos | 89,756 | Mexico | 46.2±16.0 | 43.6 | Incidence, hospitalization, mortality | Medical record |  | Cross-sectional |
| Goyal (a) | 393 | USA (NY) | 48.6-73.7 | 39.4 | IMV admission | Medical record |  | Cross-sectional |
| Goyal (b) | 1,687 | USA (NY) | 53.7-77.2 | 40.5 | Hospitalization | Medical record |  | Cohort |
| Gu | 5,698 | USA (MI) | 47.3 ±20.9 | 62 | Incidence, hospitalization, mortality | Medical record |  | Cohort |
| Hajifathalian | 770 | USA (NY) | 64.0±16.7 | 39.2 | ICU admission, mortality | Medical record |  | Cross-sectional |
| Halasz | 242 | Italy | 56-71 | 18.2 | Mortality | Medical record |  | Cross-sectional |
| Hamer | 760 | UK | 56.2 ± 8.0 | 55.1 | Hospitalization | Medical record |  | Cohort |
| Hernández-Garduño | 12,304 | Mexico | 38-59 | 41.3 | Incidence | Medical record | Age, gender | Case-control |
| Ho | 340 | UK | 8.5 | 42.9 | Incidence | Medical record | Age, gender | Cohort |
| Hu | 323 | China | 23-91 | 48.6 | Severity of disease, mortality | Medical record |  | Cross-sectional |
| ICARNS | 10,421 | UK | 12.7 | 29.7 | Incidence, hospitalization, mortality | Self-reported |  | Cross-sectional |
| Kaeuffer | 1,045 | France | 66.3±16.0 | 41.4 | Severity of disease | Medical record |  | Cohort |
| Kalligeros | 103 | USA (MD) | 52-70 | 48.5 | ICU admission | Medical record | Age, gender, race, and other | Retrospective cohort |
| Kebisek | 219 | USA | Adults, 18+ | 20.1 | Incidence, hospitalization | Medical record |  | Cross-sectional |
| Khawaja | 605 | UK | 54.7±8.8 | 43.1 | Incidence, hospitalization | Medical record | Age, gender, race, and other | Case-control |
| Kim | 2,491 | USA | 50-75 | 46.8 | Hospitalization, ICU admission | Medical record | Age, gender, race, and other | Cross-sectional |
| Leal | 444 | Brazil | 10 y and older | 55 | Hospitalization | Self-reported |  | Cross-sectional |
| Leung |  | Global | 0+ | Not reported | Mortality | Medical record | Age, gender, income, and other | Cross-sectional |
| Li | 182 | China | 68.5±8.8 | 64.3 | Incidence | Self-reported |  | Cross-sectional |
| Liao | 81 | China | 39.0 - 65.0 | 37 | Mortality | Self-reported |  | Cohort |
| Lighter | 3,615 | USA | Not reported | Not reported | Hospitalization | Medical record |  | Cross-sectional |
| Liu | 30 | China | 35±8 | 66.7 | Severity of disease | Self-reported |  | Cross-sectional |
| Mendy | 689 | USA (OH) | 35.2-67.5 | 47 | Hospitalization, ICU admission, mortality | Medical record |  | Cohort |
| Merzon | 782 | Israel | 35.6±0.6 | 50.8 | Incidence | Medical record |  | Cross-sectional |
| Murillo-Zamora | 5,393 | Mexico | 18+ | 36.4 | Mortality | Medical record |  | Cohort |
| Ortiz-Brizuela | 309 | Mexico | 33-54 | 40.8 | Hopitalization, ICU admission | Medical record |  | Cohort |
| Palaiodimos | 200 | USA (NY) | 50-73.5 | 51 | IMV admission, admission | Medical record |  | Cross-sectional |
| Peng | 112 | China | 55-67 | 52.7 | Severity of disease | Self-reported |  | Cross-sectional |
| Petrilli | 4,103 | USA (NY) | 36-65 | 49.5 | Hospitalization, mortality | Medical record |  | Cross-sectional |
| Pettit | 238 | USA (IL) | 58.5±17.0 | 52.5 | Mortality | Medical record | Age, gender | Cohort |
| Pongpirul | 193 | Thailand | 29-53 | 41.5 | Severity of disease | Medical record |  | Cross-sectional |
| Prats-Uribe | 1,039 | UK | 37-73 | 46.2 | Incidence | Medical record |  | Cohort |
| Qi | 267 | China | 20-80 | 44.2 | Severity of disease | Self-reported |  | Cross-sectional |
| Raisi-Estabragh | 669 | UK | 69±8.7 | 43.5 | Incidence | Medical record |  | Cross-sectional |
| Regina | 200 | Switzerland | 55-81 | 40 | IMV admission | Medical record |  | Cross-sectional |
| Reilev | 9,519 | Denmark | 34-63 | 57.9 | Hopitalization, ICU admission | Measured | Age, gender, and other | Cohort |
| Reyes Gil | 217 | USA (NY) | Adults, 18+ | 41.9 | Mortality | Medical record |  | Cross-sectional |
| Rossi | 95 | Italy | 62.5±11.8 | 38.9 | Mortality | Measured | Age, gender | Cohort |
| Sainaghi | 1,697 | Italy | 58-80 | 41 | Mortality | Self-reported |  | Cohort |
| Sapey | 2,217 | UK | 58-84 | 41.8 | Mortality | Self-reported |  | Cohort |
| Shah | 522 | USA (GA) | 50-72 | 58.2 | Mortality | Medical record |  | Cross-sectional |
| Simonnet | 124 | France | 51-70 | 27.4 | IMV admission | Medical record |  | Cross-sectional |
| Singh | 4,289 | USA | 50.2 ± 15.6 | 62.9 | Mortality | Medical record |  | Case-control |
| Sisó-Almirall | 322 | Spain | 56.7±17.8 | 50 | Hospitalization, ICU admission, mortality | Medical record |  | Cross-sectional |
| Souza | 4,826 | Brazil | 0+ | 53.8 | Mortality | Medical record |  | Cohort |
| Steinberg | 210 | USA (NJ) | 18-45 | Not reported | Hospitalization, IMV admission, mortality | Medical record |  | Cohort |
| Stoian | 432 | Romania | 67±13.1 | 34.7 | Severity of disease | Medical record |  | Cross-sectional |
| Suleyman | 463 | USA (MI) | 57.5±16.8 | 55.9 | Hospitalization, ICU admission | Medical record |  | Cohort |
| Trecarichi | 50 | Italy | 80±12 | 52 | Mortality | Medical record |  | Cross-sectional |
| Vaquero | 146 | Spain | 57.8-71 | 32.2 | ICU admission | Medical record |  | Case-control |
| Wang | 58 | USA (NY) | 67±12.5 | 48.3 | Mortality | Medical record |  | Cohort |
| Wu | 280 | China | 43.1±19 | 46.1 | Severity of disease | Medical record |  | Cohort |
| Yadaw | 3,841 | USA | 56.2±19 | 44.7 | Mortality | Medical record |  | Cohort |
| Yanover | 4,353 | Israel | 22-54 | 44.5 | Severity of disease | Medical record |  | Cohort |

**Table 3. Association between being obese and the risk of COVID-19**

| **First author** | **N** | | **% obesity** | | | | **OR** | **p** | |  |
| --- | --- | --- | --- | --- | --- | --- | --- | --- | --- | --- |
|  |  |  | **Non-COVID-19** | | **COVID-19** | |  |  |  |  |
| Reilev | 9,519 | 9.9 | | 8.6 | | 0.86 (0.80 – 0.93) | | | <0.0001 | |
| Alam | 1,516 | 35.2 | | 33.9 | | 0.94 (0.84 – 1.05) | | | 0.28 | |
| Merzon | 782 | 29.7 | | 33.1 | | 1.17(0.99 – 1.38) | | | 0.061 | |
| Burn | 109,367 | 16.6 | | 20.6 | | 1.30 (1.12 – 1.32) | | | <0.0001 | |
| Hernández-Garduño | 12,304 | 32.4 | | 38.5 | | 1.31 (1.25 – 1.37) | | | <0.0001 | |
| Antonio-Villa | 10,925 | 15.0 | | 18.7 | | 1.31 (1.23 – 1.39) | | | <0.0001 | |
| Denova‐Gutiérrez | 3,844 | 12.8 | | 17.4 | | 1.44 (1.31 – 1.58) | | | <0.0001 | |
| Cho | 538 | 28.5 | | 33.6 | | 1.48 (1.14 – 1.92) | | | .00031 | |
| Stoian | 432 | 8.9 | | 13.0 | | 1.53 (1.05 – 2.24) | | | 0.028 | |
| Giannouchos | 89,756 | 14.3 | | 20.5 | | 1.54 (1.51 – 1.58) | | | <0.0001 | |
| Bello-Chavolla | 15,529 | 14.0 | | 20.7 | | 1.61 (1.53 - 1.68) | | | <0.0001 | |
| Berumen | 31,522 | 14.2 | | 21.0 | | 1.61 (1.55 – 1.66) | | | <0.0001 | |
| Darling | 580 | 33.2 | | 40.2 | | 1.67 (1.24 - 2.26) | | | <0.0001 | |
| de Lusignan | 587 | 24.5 | | 31.3 | | 1.68 (1.33 – 2.13) | | | <0.0001 | |
| ICARNS | 10,421 | 47.1 | | 60.7 | | 1.73 (1.59 – 1.88) | | | <0.0001 | |
| Ho | 340 | 23.3 | | 34.1 | | 1.97 (1.46 - 2.65) | | | <0.0001 | |
| Khawaja | 605 | 23.3 | | 34.2 | | 2.23 (1.79 – 2.77) | | | <0.0001 | |
| Gu | 5,698 | 57.8 | | 76.3 | | 2.35 (1.94 – 2.85) | | | <0.0001 | |
| Gao | 150 |  | |  | | 2.91 (1.31 – 6.47) | | | .007 | |
| **Pooled Data** | **304,415** |  | |  | | **1.46 (1.30 – 1.65)** | | | **<0.0001** | |

**Table 4. Prevalence of overweight/obesity and risk of hospitalized of COVID-19 patients**

| **First author** | **N** | **% obesity** | | **OR** | **p** |
| --- | --- | --- | --- | --- | --- |
|  |  | **Non-hospitalized** | **Hospitalized** |  |  |
| Singh | 4,289 | 48.9 | 53.0 | 1.18 (1.04 – 1.34) | 0.011 |
| Lighter | 3,615 | 33.5 | 41.1 | 1.38 (1.20 - 1.60) | <0.0001 |
| Giannouchos | 89,756 | 18.6 | 24.1 | 1.39 (1.34 – 1.44) | <0.0001 |
| Berumen | 31,522 | 18.4 | 24.8 | 1.46 (1.39 – 1.55) | <0.0001 |
| Suleyman | 463 | 48.2 | 59.2 | 1.56 (1.01 – 2.40) | 0.043 |
| Carrillo-Vega | 10,544 | 17.7 | 25.2 | 1.56 (1.42 – 1.72) | <0.0001 |
| Reilev | 9,519 | 7.7 | 12.1 | 1.66 (1.42 – 1.94) | <0.0001 |
| Mendy | 689 | 15.9 | 24.5 | 1.73 (116 – 2.56) | 0.0066 |
| Gu | 5689 | 71.2 | 82.4 | 1.90 (1.31 – 2.74) | 0.0006 |
| Ebinger | 442 | 11.8 | 20.6 | 2.04 (1.14 - 3.65) | 0.02 |
| Hamer | 760 | 23.5 | 36.4 | 2.28 (1.88 – 2.77) | <0.0001 |
| Steinberg | 210 | 37.1 | 60.6 | 2.62 (1.49 – 4.58) | 0.0007 |
| Yanover | 4,353 | 19.2 | 42.2 | 3.08 (2.26 – 4.20) | <0.0001 |
| Sisó-Almirall | 322 | 7.9 | 20.9 | 3.09 (1.56 – 6.12) | <0.0001 |
| Burn | 109,367 | 6.7 | 18.2 | 3.09 (2.95 – 3.23) | <0.0001 |
| Petrilli | 4,103 | 14.4 | 39.8 | 3.92 (3.37 - 4.56) | <0.0001 |
| Ortiz-Brizuela | 309 | 10.1 | 35.7 | 4.97 (2.70 – 9.13) | <0.0001 |
| Kebisek | 219 | 21.7 | 58.3 | 5.04 (1.53 – 16.6) | <0.0001 |
| Leal | 444 | 38.3 | 81.8 | 7.27 (2.38 – 22.2) | <0.0001 |
| **Pooled data** | **276,615** |  |  | **2.13 (1.74 – 2.60)** | **<0.0001** |

**Table 5. The association between obesity and ICU admission**

| **First author** | **N** | **% Obesity** | | **OR** | **p** |
| --- | --- | --- | --- | --- | --- |
|  |  | **Not admitted ICU** | **Admitted ICU** |  |  |
| Reilev | 9,519 | 12.1 | 12.0 | 0.99 (0.68 – 1.44) | 0.95 |
| Suleyman | 463 | 57.5 | 61.7 | 1.19 (0.77 – 1.84) | 0.43 |
| Vaquero | 146 | 29.3 | 33.8 | 1.23 (0.61 – 2.48) | 0.56 |
| Ebinger | 442 | 19.7 | 22.1 | 1.26 (0.62 - 2.57) | 0.52 |
| Bello-Chavolla | 15,529 | 10.5 | 13.2 | 1.29 (1.15 - 1.45) | <0.0001 |
| Kim | 2,491 | 47.4 | 54.5 | 1.33 (1.12 – 1.58) | 0.0013 |
| Pettit | 238 | 73.5 | 79.2 | 1.37 (0.62 – 3.01) | 0.43 |
| Feuth | 28 | 35.0 | 42.9 | 1.39 (0.24 – 8.07) | 0.71 |
| Gu | 5698 | 79.6 | 85.3 | 1.48 (0.82 – 2.67 | 0.19 |
| Lighter | 3,615 | 36.7 | 46.9 | 1.52 (1.24 - 1.86) | 0.0035 |
| Cai | 383 | 10.3 | 14.3 | 1.62 (0.56 - 4.71) | 0.37 |
| Kalligeros | 103 | 40.6 | 56.8 | 1.63 (0.67 - 3.94) | 0.28 |
| Caussy (a) | 110 |  |  | 1.69 (1.10 – 2.59) | 0.0011 |
| Mendy | 689 | 17.4 | 26.4 | 1.70 (1.02 – 2.84) | 0.04 |
| Hajifathalian | 770 | 33.6 | 48.2 | 1.84 (1.32 – 2.57) | 0.0003 |
| Kaeuffer | 1,045 | 52.5 | 71.0 | 2.21 (1.56 – 3.15) | <0.0001 |
| Pongpirul | 193 | 15.8 | 30.0 | 2.28 (0.76 – 6.81) | 0.13 |
| Ortiz-Brizuela | 309 | 31.5 | 51.7 | 2.33 (1.01 – 5.34) | 0.04 |
| Caussy (b) | 124 |  |  | 2.86 (1.68 – 4.71) | 0.0017 |
| Al-Sabah | 1,158 | 20.3 | 33.8 | 3.51 (1.73 – 7.12) | 0.0003 |
| Qi | 267 | 7.4 | 28.0 | 4.89 (2.19 – 10.87) | <0.0001 |
| Gerotziafas | 310 | 7.7 | 31.4 | 5.48 (2.84 – 10.61) | <0.0001 |
| **Pooled data** | **43,630** |  |  | **1.74 (1.46 - 2.08)** | **<0.0001** |

**Table 6. The association between obesity and IMV admission**

| First author | N | % Obesity | | OR | p |
| --- | --- | --- | --- | --- | --- |
|  |  | Non-IMV | IMV |  |  |
| Hajifathalian | 770 | 33.7 | 37.5 | 1.18 (0.86 – 1.61) | 0.31 |
| ICARNS | 10,421 | 57.7 | 62.0 | 1.20 (1.07 – 1.33) | 0.0013 |
| Bello-Chavolla | 15,529 | 10.3 | 13.1 | 1.31 (1.17 – 1.48) | <0.0001 |
| Ebinger | 442 |  | 16.1 | 1.57 (0.72 – 3.41) | 0.26 |
| Caussy | 291 | 26.9 | 37.2 | 1.61 (0.97 – 2.68) | 0.066 |
| Cai | 383 | 10.3 | 14.3 | 1.62 (0.56 – 4.71) | 0.37 |
| Pettit | 238 | 73.7 | 82.1 | 1.65 (0.59 – 4.59) | 0.34 |
| Goyal | 1,687 | 45.2 | 59.4 | 1.78 (1.35 – 2.34) | <0.0001 |
| Singh | 4,289 | 49.3 | 63.9 | 1.82 (1.39 – 2.38) | <0.0001 |
| Regina | 200 | 23.3 | 43.2 | 2.51 (1.19 – 5.28) | 0.014 |
| Kalligeros | 103 | 40.5 | 65.5 | 2.79 (1.14 – 6.82) | 0.022 |
| Simonnet | 124 | 28.2 | 56.5 | 3.30 (1.46 – 7.49) | 0.0034 |
| Steinberg | 210 | 40.8 | 80.6 | 6.01 (2.50 – 14.48) | <0.0001 |
| **Pooled data** | **36,374** |  |  | **1.66 (1.38 - 1.99)** | **<0.0001** |

**Table 7. The association between obesity and prognosis of COVID-19**

| **First author** | **N** | **% Obesity** | | **OR** | **p** |
| --- | --- | --- | --- | --- | --- |
|  |  | **Discharge alive** | **Dead** |  |  |
| Trecarichi | 50 | 11.8 | 7.1 | 0.58 (0.06 – 5.67) | 0.63 |
| Goyal | 1,687 | 51.4 | 38.5 | 0.59 (0.43 – 0.81) | 0.0009 |
| Hajifathalian | 770 | 38.5 | 27.9 | 0.62 (0.37 – 1.03) | 0.06 |
| Shah | 522 | 67.4 | 56.5 | 0.63 (0.40 – 0.99) | 0.046 |
| Gu | 5,689 | 82.9 | 76.9 | 0.69 (0.26 – 1.80) | 0.30 |
| Kim | 2,491 | 51.2 | 42.0 | 0.69 (0.55 – 0.86) | 0.0009 |
| ICARNS | 10,421 | 41.4 | 36.6 | 0.82 (0.74 - 0.92) | 0.0003 |
| Petrilli | 4,103 | 42.9 | 42.0 | 0.97 (0.78 - 1.19) | 0.74 |
| Sapey | 2,217 | 11.2 | 11.5 | 1.02 (0.75- 1.40) | 0.88 |
| Reyes Gil | 217 |  |  | 1.04 (0.98 - 1.04) | 0.18 |
| Singh | 4289 | 49.9 | 52.3 | 1.10 (0.81 – 1.49) | 0.54 |
| Yadaw | 3,841 | 5.8 | 7.3 | 1.28 (0.82 – 2.00) | 0.28 |
| Argenziano | 1,000 | 39.5 | 45.7 | 1.29 (0.95 – 1.76) | 0.10 |
| Docherty | 16,749 |  |  | 1.37 (1.16 - 1.63) | <0.0001 |
| Murillo-Zamora | 5,393 | 20.2 | 26.4 | 1.42 (1.24 – 1.62) | <0.0001 |
| Liao | 81 | 19.7 | 10.0 | 1.45 (0.14 - 15.21) | 0.76 |
| Reilev | 9,519 | 12.8 | 10.1 | 1.50 (1.10 – 2.10) | <0.01 |
| Borobia | 2,226 | 10.0 | 14.3 | 1.51 (1.12 - 2.05) | 0.0007 |
| Baqui | 11,321 | 3.5 | 5.4 | 1.59 (1.25 – 1.96) | <0.0001 |
| Halasz | 242 | 60.4 | 39.6 | 1.61 (0.65 – 3.99) | 0.30 |
| Wang | 58 | 38.1 | 50.0 | 1.63 (0.41 – 6.39) | 0.49 |
| Pettit | 238 | 74.1 | 82.4 | 1.63 (0.45 – 5.92) | 0.46 |
| Sainaghi | 1697 | 20.3 | 30.9 | 1.75 (0.94 – 3.27) | 0.07 |
| Carrillo-Vega | 10,544 | 19.6 | 30.0 | 1.75 (1.51 – 2.03) | <0.0001 |
| Bello-Chavolla | 15,529 | 6.7 | 30.3 | 1.77 (1.57 - 1.99) | <0.0001 |
| Gabazzi | 279 | 8.0 | 15.0 | 2.08 (0.96 - 4.51) | 0.065 |
| Souza | 4,826 | 4.9 | 9.9 | 2.14 (1.49 – 3.07) | <0.0001 |
| Giacomelli | 233 | 13.5 | 27.1 | 2.38 (1.11 – 5.10) | 0.023 |
| Palaiodimos | 200 |  |  | 2.56 (1.18 – 5.57) | 0.018 |
| Giannouchos | 89,756 | 18.0 | 40.5 | 3.10 (2.98 – 3.23) | <0.0001 |
| Rossi | 95 | 48.2 | 69.2 | 3.47 (1.00 – 12.03) | 0.04 |
| Hu | 323 | 2.9 | 10.7 | 3.96 (1.28 - 12.29) | 0.01 |
| Burn | 109,367 | 14.2 | 34.0 | 4.03 (3.69 – 4.40) | <0.0001 |
| Steinberg | 210 | 44.3 | 83.3 | 6.29 (1.76 – 22.45) | 0.0015 |
| Antwi-Amoabeng | 172 |  |  | 10.55 (1.07 – 104.45) | 0.04 |
| Peng | 112 | 18.9 | 88.2 | 32.08 (6.73 - 153.00) | <0.0001 |
| **Pooled data** | **316,467** |  |  | **1.48 (1.22 – 1.80)** | **<0.0001** |
